# The role of inflammatory cells in fostering pancreatic cancer cell growth and invasion

**DOI:** 10.3389/fphys.2012.00270

**Published:** 2012-07-13

**Authors:** Anthony Evans, Eithne Costello

**Affiliations:** ^1^Liverpool Cancer Research UK Centre, University of LiverpoolLiverpool, UK; ^2^The National Institute for Health Research Pancreas Biomedical Research Unit, Department of Molecular and Clinical Cancer Medicine Centre, University of LiverpoolLiverpool, UK

**Keywords:** mast cells, myeloid-derived suppressor cells, neutrophils, regulatory T cells, T helper cells, macrophages, inflammation, stroma

## Abstract

The pancreatic ductal adenocarcinoma (PDAC) microenvironment accommodates a variety of cell types and a plethora of complex interactions between tumor cells, host cells and extracellular matrix (ECM) components. Here we review the role of inflammatory cells, in particular mast cells, myeloid-derived suppressor cells, macrophages, T regulatory cells, T helper cells and neutrophils. The picture that emerges is that of a tumor microenvironment, in which the immune response is actively suppressed, and inflammatory cells contribute in a variety of ways to tumor progression.

## Introduction

The presence of dense desmoplastic stroma is one of the defining characteristics of PDAC (Neesse et al., 2010). PDAC stroma consists of mesenchymal cells such as fibroblasts and pancreatic stellate cells (PSCs); ECM proteins including proteoglycans, fibronectin, and collagens I and III; peri-tumoral nerve fibers; endothelial cells and cells of the immune system (Korc, 2007; Farrow et al., 2008). The resulting microenvironment contributes to cancer initiation, tumor progression (Hwang et al., 2008; Vonlaufen et al., 2008; Whiteside, 2008; Polyak et al., 2009) and chemoresistance (Olive et al., 2009; Grippo and Tuveson, 2010).

Inflammatory cells have been linked to tumor development and progression through their mediation of the inflammatory response critical for tumor formation from precancerous lesions (de Visser et al., 2006). In the case of PDAC, a notably higher incidence of cancer is found in patients with the inflammatory condition, chronic pancreatitis (CP; Lowenfels et al., 1993; McKay et al., 2008). Moreover for individuals with hereditary pancreatitis, the cumulative lifetime risk (to 70 years of age) of pancreatic cancer is very high, at 40 (Vitone et al., 2005). In genetically modified mouse models of PDAC, cerulein-induced pancreatitis is known to accelerate the development of PDAC (Guerra et al., 2007, 2011; Morris et al., 2010). Moreover, pancreatitis may also hasten the process of metastasis (Rhim et al., 2012). Using a mouse model of pancreatic cancer in which pancreatic epithelial cells could be tracked, Rhim et al. (2012) recently made the surprising observation that pancreatic cells, in the early stages of transformation, entered the bloodstream. Interestingly, the number of circulating pancreatic cells was increased in mice following the induction of pancreatitis, and treatment with the anti-inflammatory, dexamethasone, blocked dissemination.

The relationship between tumor cells and the immune system is complex. Infiltrations of cells from both the innate and the adaptive immune systems have been observed in many tumor types and, depending on their nature, have been linked to prognostic outcome (Pages et al., 2010; Roxburgh and McMillan, 2011) and response to treatment with conventional chemotherapies (Fridman et al., 2011). One of the hallmarks of cancer is the need for tumors to evade immune destruction (Hanahan and Weinberg, 2011), and yet equally important in many cases is the necessity of the tumor to hijack inflammatory components of the immune response to create an environment that fosters tumor growth and progression (Farrow et al., 2008; Hanahan and Weinberg, 2011).

**Figure 1:**
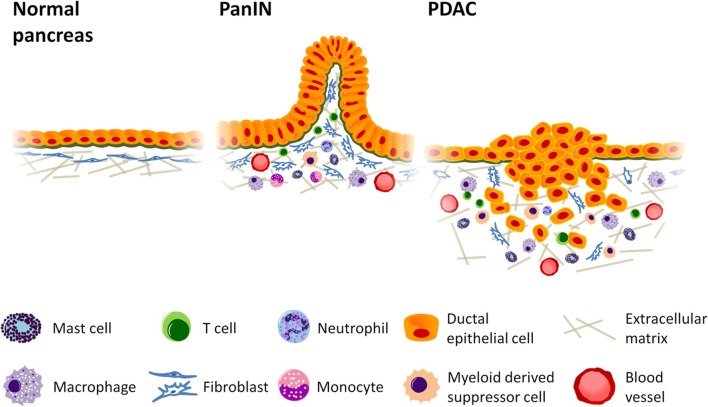
**Inflammatory cell infiltration promotes growth and invasion in pancreatic cancer.** Desmoplastic stroma accumulates as tumorigenesis progresses, increasing vascular formation and the production of collagen while recruiting cells of the immune system to enhance tumor growth. Mast cells and activated tumor associated macrophages (TAMs) localize at the leading edge of the tumor where they can accelerate tumor invasion, and TAMs are thought to enhance lymphatic metastasis and angiogenesis. Myeloid derived suppressor cells (MDSCs), likewise, accumulate at the invasive front and inhibit CD8^+^ cytotoxic T cells, enabling immune evasion. Regulatory T cells (Tregs), which also suppress immune function, can be found in relatively high abundance in pancreatic ductal adenocarcinoma (PDAC). The presence of Th2 T helper cells in greater numbers than Th1 has also been linked to increased tumor growth. PanIN = Pancreatic intraepithelial neoplasia.

In this review we focus on the inflammatory components of pancreatic stroma that contribute to immune suppression, tumor cell growth and invasion. The negative growth effects of cellular components, such as cytotoxic T lymphocytes, natural killer (NK) and dendritic cells, along with their potential exploitation in the development of immunotherapies are reviewed elsewhere (Koido et al., 2011; Vanneman and Dranoff, 2012; Wachsmann et al., 2012). The role of specified inflammatory cells in PDAC are detailed below and summarized in both Figure 1 and Table 1.

**Table 1:** **Summary of the role of specified inflammatory cells in pancreatic cancer**.

**Inflammatory cell**	**Relevance to PDAC**	**Reference**
Mast cell (MC)	• MC counts in human PDAC cancer edge linked to poor prognosis	Cai et al., 2011
	• MCs observed at the infiltrating edges of pancreatic tumors in mice	Chang et al., 2011
	• PDAC tumor growth reduced and survival increased in MC-deficient mice	
Myeloid-derived suppressor cell (MDSC)	• MDSC levels are elevated in peripheral blood of cancer patients (including PDAC patients) compared to controls. The risk of death was increased as the percentage of MDSCs increased	Gabitass et al., 2011
	• MDSCs infiltration of pancreatic tumors in genetically modified mice is accompanied by a lack of T cells, especially CD8^+^ cytotoxic T cells	Clark et al., 2007
Tumor associated macrophage (TAM)	• M2-polarised TAMs (CD163 or CD204-positive macrophages), but not CD68- positive macrophages, associated with a poor prognosis linked to accelerated lymphatic metastasis	Kurahara et al., 2011
	• SPARC positively influences the infiltration and the distribution of macrophages to pancreatic tumors in mice	Puolakkainen et al., 2004
	• TAMs recruited to orthotopic pancreatic tumors in mice express VEGF receptor 2, which is involved in macrophage recruitment to these tumors	Dineen et al., 2008
	• The interaction of macrophage inflammatory protein-3α and chemokine receptor 6 (CCR6) is proposed to increase PDAC cell proliferation, migration and invasion	Kimsey et al., 2004; Campbell et al., 2005
	• CD40 activation in pancreatic cancer re-established tumor immune surveillance by targeting macrophages, resulting in the destruction of the tumor stroma	Kleeff et al., 1999; Beatty et al., 2011
T cell	• Regulatory T cells (Tregs) are elevated in the blood of PDAC patients and correlated with the numbers of MDSCs	Gabitass et al., 2011
	• Tregs numbers increase with PanIN progression and advancing grade of PDAC	Hiraoka et al., 2006
	• Elevated ratio of Th2 to Th1 T helper cells is associated with poor prognosis in PDAC	de Monte et al., 2011
Neutrophil	• Significant tumor-infiltrating neutrophils are uncommon in PDAC	Reid et al., 2011

Only a selection of published data are presented here.

## Mast cells

Mast cells are associated with allergic and anaphylactic reactions and contribute to immune responses to microbial infection (Gilfillan and Beaven, 2011). Other functions include facilitating tissue remodeling and repair following injury, tumor repression and tumor growth (Gilfillan and Beaven, 2011). Mast cells produce and secrete potent angiogenic molecules, and have been implicated in angiogenesis in various malignancies, including laryngeal squamous cell carcinomas, lung cancers and malignant melanomas (Norrby, 2002). As tumors develop, mast cell precursors are attracted to the tumor stroma. They express the receptor for stem cell factor (KIT) and tumor-derived stem cell factor is believed to cause the recruitment and activation of mast cells in tumors (Ribatti and Crivellato, 2011). Once activated, mast cells release inflammatory factors such as interleukin-6 (IL-6), tumor necrosis factor-α, and vascular endothelial growth factor (VEGF; Ribatti and Crivellato, 2011). Through the secretion of immunosuppressive cytokines such as IL-10, mast cells downregulate the immune response to tumors (Ribatti and Crivellato, 2011). They also favor expansion and activation of regulatory T (Treg) cells which promote immune tolerance (Ribatti and Crivellato, 2011).

In a transgenic mouse model of pancreatic cancer which expresses high levels of mutant K-ras (*KRas*^*G*12*V*^), PDAC was associated with an influx of mast cells (Chang et al., 2011). In this model, mast cells were evenly distributed in CP and pancreatic intraepithelial neoplasia (PanIN) lesions, whereas they were observed at the infiltrating edges of tumor. To determine the effects of mast cells on PDAC growth, pancreatic tumors were grown orthotopically in mast cell-deficient mice (Kit^−/−^ mice). PDAC growth was significantly reduced in Kit^−/−^ mice compared to control mice, and the mast cell-deficient mice lived longer. These findings matched those in human tissues from patients with PDAC (*n* = 67), in which higher levels of mast cells corresponded with poor outcome, corroborating a trend seen in a previous study (Esposito et al., 2004). More recently, high mast cell counts in the cancerous border with normal tissue were linked to poor prognosis (Cai et al., 2011). *In vitro* studies have indicated that there may be cross-talk between mast cells and PDAC cells. One study noted that signals secreted from PDAC cells stimulated mast cell migration whilst reciprocating signals selectively increased proliferation of tumor cells (Strouch et al., 2010). Furthermore mast cells were found to promote the invasiveness of pancreatic tumor cells in a matrix metalloproteinase (MMP)-dependent manner (Strouch et al., 2010).

The observation of mast cell infiltration in CP and PanIN lesions in *KRas*^*G*12*V*^ mice (Chang et al., 2011), suggests that recruitment of mast cells may occur early in the development of pancreatic cancer and is consistent with the notion of inflammation potentiating neoplasia. It is likely that mast cells are involved in signaling with other components of the tumor microenvironment as part of the overall inflammatory response. IL-33 is known to activate mast cells and stimulate pro-inflammatory cytokine production (Xu et al., 2008), and has been found to be expressed in the nuclei of activated PSCs (Masamune et al., 2010). Similarly, mast cell tryptase contained within mast cell granules and released upon degranulation has been found to stimulate hepatic stellate cell proliferation and collagen production, an essential process in stromal formation (Gaça et al., 2002). With the notable presence of mast cells in the stroma of PDAC it will be interesting to elucidate their full contribution to the pancreatic cancer tumor microenvironment and to PDAC cells directly.

## Myeloid-derived suppressor cells

A number of different myeloid-derived cells feature within tumor stroma, including myeloid-derived suppressor cells (MDSCs), tumor-associated macrophages (TAMs), and dendritic cells. These cells and their ability to suppress the tumoral immune response, either alone or through communication with each other, are reviewed by Ostrand-Rosenberg et al. (2012). MDSCs are immature myeloid cells that enhance tumor growth by promoting angiogenesis and by suppressing components from both the innate and the adaptive immune system (Ostrand-Rosenberg and Sinha, 2009; Ochando and Chen, 2012). They are elevated in both the circulation and the tumor microenvironment of patients with cancer, and comprise two main subsets, a monocytic subpopulation expressing CD14 and a granulocytic subpopulation expressing CD15 (Ostrand-Rosenberg et al., 2012). MDSCs use a variety of mechanisms to actively suppress host immunity such as inhibition of T-cell activation, through the production of reactive oxygen (Kusmartsev et al., 2004) and nitrogen species and the depletion of the amino acids arginine and L-cysteine; inhibition of T cell migration; expansion of immunosuppressive Tregs and inhibition of NK cell cytotoxicity (Ostrand-Rosenberg et al., 2012).

In a genetically modified mouse model of pancreatic cancer, in which oncogenic *KRas*^*G*12*D*^ is expressed in a pancreas-specific fashion, the analysis of immune cells during pancreatic cancer progression (Clark et al., 2007) revealed a slight elevation in the number of MDSCs in PanIN lesions, giving way to a more pronounced increase in PDAC. MDSCs accumulated around periductal areas and stroma in PDAC, although their infiltration was delayed compared to macrophages (Clark et al., 2007). Interestingly, the presence of MDSC infiltrates was accompanied by a lack of T cells, especially CD8^+^ cytotoxic T cells. This is consistent with previous work which demonstrated that MDSCs inhibit the CD8^+^ T cell response through the production of reactive oxygen species (Kusmartsev et al., 2004). The inhibitory effect of MDSCs on CD8^+^ T cells was further supported by a recent study in which murine pancreatic cancer cells (Panc02) were inoculated into immunocompetent mice (Pilon-Thomas et al., 2011). Tumor-bearing mice exhibited down-regulation of src homology 2 domain-containing inositol 5’-phosphatase-1 (SHIP-1) expression in splenocytes, and an expansion of MDSCs in the peripheral blood and splenocytes. MDSCs from tumor-bearing mice overexpressed Bcl-2, contained hyper-phosphorylated Akt (Pilon-Thomas et al., 2011) and were found to suppress CD8^+^ T cell growth to a greater extent than MDSCs from control mice (Pilon-Thomas et al., 2011).

In a study of patients with pancreatic (*n* = 46), oesophageal (*n* = 60) and gastric (*n* = 25) cancers, both Treg and MDSC (HLADR^−^ Lin1^*low*/−^ CD33^+^ CD11b^+^) levels were found elevated in peripheral blood of cancer patients compared to 54 healthy controls (Gabitass et al., 2011). The risk of death was increased as the percentage of MDSCs increased (Gabitass et al., 2011).

Recently, a mechanism of tumor invasion has been identified where MDSCs alter the structure of MHC class I expressed on the tumor cell surface, preventing the binding of processed peptide that would otherwise activate antigen-specific CD8^+^ T cells (Lu et al., 2011). This process was linked to the production of peroxynitrite (PNT), the predominant source of which, in pancreatic cancer patients, was myeloid cells.

## Macrophages

Macrophages normally fight infection, facilitate wound healing and regulate tissue homeostasis. They localize to sites of inflammation by responding to the chemoattractant, monocyte chemotactic protein 1 (MCP-1; Farrow et al., 2008), and act as one of the crucial effectors of the inflammatory response, releasing cytokines, and growth factors (Coussens and Werb, 2002). Their presence in the microenvironment of tumors has prompted analysis of their role in cancer (reviewed in Ostrand-Rosenberg et al., 2012; Ruffell et al., 2012). TAMs possess a number of characteristics that facilitate tumor growth and support metastasis. Through the production of vascular endothelial growth factor A (VEGFA), they contribute to angiogenesis; by producing components of ECM, such as collagens and ECM degrading enzymes, such as MMPs, they contribute to stromal remodeling and to cell migration within the tumor (Ostrand-Rosenberg et al., 2012; Ruffell et al., 2012). Macrophages are classified into two extreme phenotypic types, pro-inflammatory M1 macrophages and anti-inflammatory M2 macrophages, although in reality the phenotype of macrophages is complex and cannot be fully explained by a simple division into two discrete groups. M1-like macrophages promote tumor cell death, while M2-like macrophages favor tumor progression (Ostrand-Rosenberg et al., 2012; Ruffell et al., 2012).

An assessment of TAMs in PDAC tumors, using the common macrophage marker, CD68 as well as markers specific for receptors on M2-polarised macrophages, CD163 and CD204, found that the presence of high numbers of M2-polarised macrophages correlated with a high incidence of lymph node metastasis. Moreover, high lymphatic vessel density, as measured using D2–40, and poor prognosis were observed in cases with high number of CD163 or CD204-positive macrophages, but not CD68-positive macrophages (Kurahara et al., 2011).

A number of mechanisms have been proposed for the recruitment and activity of macrophages in pancreatic tumors. Macrophage inflammatory protein-3α (Mip-3α) is expressed in PDAC and mononuclear inflammatory cells in PDAC patient tissues (Kimsey et al., 2004; Campbell et al., 2005). Specific interactions of Mip-3α and its receptor, chemokine receptor 6 (CCR6) which is expressed in PDAC cells, have been shown to increase PDAC cell proliferation, migration and invasion in type IV collagen, possibly through upregulation of MMP-9 production in the tumor cells (Kleeff et al., 1999; Kimsey et al., 2004; Campbell et al., 2005). Similarly, a role for VEGF has been elucidated. VEGF is one of several cytokines to mediate macrophage recruitment to tumors. Dineen et al. (2008) demonstrated the expression of VEGF receptor 2 on TAMs of orthotopic pancreatic tumors in mice. Selective inhibition of this receptor diminished macrophage infiltration into these tumors. Focal adhesion kinase (FAK), a known regulator of cell migration, proliferation, apoptosis, and survival, has also been implicated in enabling macrophage infiltration in PDAC (Stokes et al., 2011). Interestingly, an intracellular matrix protein, the matricellular glycoprotein secreted protein acidic and rich in cysteine (SPARC) has been shown to influence the infiltration and the distribution of macrophages to pancreatic tumors in mice. Panc02 cells were grown subcutaneously in SPARC-null and wild-type SPARC mice. F4/80-expressing murine macrophages were more plentiful in tumors from wild-type mice, and were distributed at the margins of tumors. By contrast in SPARC-null mice, the distribution of macrophages was even throughout the tumors (Puolakkainen et al., 2004).

Other mechanisms underpinning the actions of macrophages in pancreatic cancer have also been suggested. TAMs were found to produce Sonic hedgehog (Shh) upon stimulation of the NF-κ B pathway, causing an increase in PDAC cell proliferation, while IL-1β release from stimulated macrophage cells was shown to protect PDAC cells from drug-induced apoptosis by upregulating cyclooxygenase 2 (COX-2) production (Angst et al., 2008; Yamasaki et al., 2010).

## T cells

CD3^+^ T lymphocyte infiltrations have been reported in both human and mouse PDAC tissue specimens (von Bernstorff et al., 2001; Clark et al., 2007). Studies have focused on the presence and function of CD4^+^ CD25^*high*^ T regulatory cells (Tregs; Hiraoka et al., 2006; Gabitass et al., 2011). Like MDSCs, Tregs display an immunosuppressive phenotype, and are important mediators of immune evasion in cancer. The mechanisms used by Tregs to suppress immune function, including the suppression of CD4^+^ CD25^−^ conventional T cells, are reviewed elsewhere (Menetrier-Caux et al., 2012; Schmidt et al., 2012). As stated already in this article, the numbers of both Tregs (CD4^+^ CD25^+^ CD127^*low*/−^ FoxP3^+^) and MDSCs were found to be elevated in the circulation of pancreatic cancer patients and correlated with one another (Gabitass et al., 2011). CD4^+^ FoxP3^+^ Tregs have been identified in PDAC tissues in significantly higher quantities than in non-neoplastic inflammatory pancreatic stroma (Hiraoka et al., 2006). Moreover, higher levels of Tregs corresponded with more poorly differentiated tumors. Similarly, analysis of progressive grades of PanIN revealed a significant increase in the prevalence of Tregs from low-grade PanIN to invasive ductal carcinoma, with a corresponding decrease in CD8^+^ T cell infiltration with PanIN progression. High tumoral Treg levels were associated with poor prognosis (Hiraoka et al., 2006).

Possible mechanisms for the increase in abundance of Tregs in the tumor microenvironment have been demonstrated in mouse models. Panc02 cells inoculated into a murine host were shown to secrete chemokine (C-C motif) ligand 5 (CCL5; Tan et al., 2009) and transforming growth factor-β (TGF-β; Moo-Young et al., 2009) resulting in the recruitment of Tregs or the conversion of CD4^+^ CD25^−^ naive T cells into Tregs (Moo-Young et al., 2009).

Along with Tregs, T helper (Th) cells have also been studied in PDAC, though the literature on the subject is limited. Th cells activate cells of the innate immune system, facilitate wound repair and provide assistance to B cells and cytotoxic CD8^+^ T cells (O'Shea and Paul, 2010; Okoye and Wilson, 2011). Several subsets of Th cell exist including Th1 cells that produce interferon-γ (IFN-γ) and induce microbial elimination, and Th2 cells that secrete IL-4 and IL-13 and are thought to mediate helminth immunity (Abbas et al., 1996; O'Shea and Paul, 2010). Immunohistochemical analyses of PDAC samples have revealed the presence of both Th1 and Th2 cell populations, with evidence that the quantity and activity of Th cells are skewed toward the Th2 subset (Bellone et al., 1999; Tassi et al., 2008). A recent study reported that the ratio of Th2 GATA-3^+^ to Th1 T-bet^+^ cells is an independent predictor of disease-free and overall survival in PDAC patients, suggesting a link between Th2 prevalence and tumor progression (de Monte et al., 2011). The authors propose a mechanism whereby tumor cells induce the release of thymic stromal lymphopoietin (TSLP) from cancer-associated fibroblasts (CAFs) which subsequently activates tumor antigen-loaded dendritic cells. They posit that dendritic cells then migrate and activate tumor antigen-specific Th2 cells at the draining lymph nodes. The activated Th2 cells then home to the tumor site and may promote tumor growth (de Monte et al., 2011). This study illustrates the important cross-talk between components of the stroma and the immune system.

## Neutrophils

Neutrophils play crucial roles in defense against infectious diseases, act as early modulators of inflammation and are often the initial recruited effector at sites of acute inflammation. They are key regulators of the immune response (Kumar and Sharma, 2010; Mantovani et al., 2011).

A recent systematic analysis of tumor infiltrating neutrophils was undertaken in a variety of pancreatic neoplasms (Reid et al., 2011). The authors defined 10 or more infiltrating neutrophils per 100 epithelial cells as positive, and classified more than 15 tumor-infiltrating neutrophils as significant. Significant neutrophil infiltrates were rarely found in PDAC, but were observed in micropapillary and undifferentiated types of poor prognosis.

In PDAC, an elevated pre-operative neutrophil-lymphocyte ratio (NLR) was reported to be associated with poor prognosis, contrasting with platelet-lymphocyte ratio, which showed no relationship with outcome (Bhatti et al., 2010). The prognostic value of NLR was verified in an independent study (Wang et al., 2012) and is consistent with observations made in other malignancies (Walsh et al., 2005; Gomez et al., 2008; Cho et al., 2009; Sarraf et al., 2009).

## Concluding remarks

Cancer cells express tumor antigens and can elicit an immune response. Unfortunately, tumors also develop mechanisms enabling themselves to escape immune surveillance. Hiraoka et al. (2011) recently investigated the host immune response in intraductal papillary mucinous neoplasm (IPMN), a precursor lesion that can lead to pancreatic cancer. The adenoma stage (IPMA) was characterized by an antitumor immune response. However, the immune reaction changed to immune tolerance in the carcinoma stage (IPMC). Inflammatory cells can contribute to tumorigenesis through immune suppression. They also facilitate cancer progression by promoting angiogenesis and facilitating cancer metastasis. Our review has focused on the tumor-promoting effects of inflammatory cells, as opposed to the anti-tumoral effects of inflammatory cells. Although good progress has been made in understanding how inflammatory cells support tumor growth, a great deal of research is still necessary before we fully appreciate the tumor promoting roles of these cells in PDAC.

### Conflict of interest statement

The authors declare that the research was conducted in the absence of any commercial or financial relationships that could be construed as a potential conflict of interest.
